# COVID-19 and Radiological Progression of Multiple Sclerosis

**DOI:** 10.3390/diagnostics16101513

**Published:** 2026-05-16

**Authors:** Hien Quang Nguyen, Roham Hadidchi, Anna Eligulashvili, Shounak Nandi, Aditi Vichare, Bhakti Patel, Jimmy Sanchez, Joseph Bisulca, Sonya Henry, Jimmy S. Lee, Tim Q. Duong

**Affiliations:** Department of Radiology, Montefiore Health System, Albert Einstein College of Medicine, New York, NY 10461, USA; hquangnguy@einsteinmed.edu (H.Q.N.); roham.hadidchi@einsteinmed.edu (R.H.);

**Keywords:** multiple sclerosis, COVID-19, neuroimaging, brain MRI, MS progression

## Abstract

**Background/Objectives**: SARS-CoV-2 infection may exacerbate neuroinflammation in patients with multiple sclerosis (MS) and thus accelerate MS progression. Previous studies have reported an increased risk of disability and lesion burden among those infected with SARS-CoV-2 while others reported no differences compared to COVID− controls. We aimed to determine whether COVID-19 is associated with accelerated radiological progression of MS. **Methods**: This single-center, retrospective longitudinal study included patients with pre-existing relapsing-remitting MS. We identified 34 SARS-CoV-2–positive MS patients (COVID+) who had at least one brain MRI prior to, and one after, their first positive PCR test. These patients were matched 2:1 by index date to 67 SARS-CoV-2–negative MS patients (COVID−). Baseline demographics and comorbidities were comparable between groups. Two radiologists independently scored pre- and post-index MRIs for new or enlarging T2 lesions, T1 gadolinium-enhancing lesions, and parenchymal brain volume loss. Logistic regression analyses evaluated group differences, adjusting for demographic and clinical covariates. **Results**: Across an average imaging interval of approximately two years, no significant differences were observed between COVID+ and COVID− patients in new lesions (8.8% vs. 9.0%), enlarging lesions (2.9% vs. 6.0%), T1-enhancing lesions (5.9% vs. 1.5%), or brain volume loss (35.3% vs. 47.8%; all *p* > 0.05). **Conclusions**: There was no detectable association between SARS-CoV-2 infection and accelerated radiological progression in patients with MS over an average two-year follow-up. Longer-term investigations are warranted to clarify whether certain subgroups or more severe COVID-19 cases might be at heightened risk.

## 1. Introduction

Multiple sclerosis (MS) is a chronic, immune-mediated disorder of the central nervous system characterized by recurring inflammatory episodes of myelin damage [1]. The underlying immunological dysfunction in MS, in addition to the use of immunomodulatory therapies such as anti-CD20 agents, makes patients with MS vulnerable to viral infections [2,3]. The COVID-19 pandemic has brought additional challenges in MS management, including risk of SARS-CoV-2 infection and its associated complications [4,5,6].

Several mechanisms have been proposed by which COVID-19 may exacerbate MS disease progression. During SARS-CoV-2 infection, a pronounced “cytokine storm” characterized by elevated interleukin-6, tumor necrosis factor-α, and interleukin-1β can intensify neuroinflammation, potentially accelerating demyelination and neurodegeneration [7,8]. This hyperinflammatory state also compromises the blood–brain barrier, permitting peripheral immune cells and cytokines to infiltrate the central nervous system and exacerbate lesion formation [9,10,11]. In addition, molecular mimicry between SARS-CoV-2 proteins and myelin antigens (e.g., proteolipid protein) may activate autoreactive T cells [12,13], and possible direct viral neuro-invasion through olfactory or hematogenous pathways could precipitate neuronal and glial damage [14]. Together, these processes can fuel microglial activation and inflammatory cascades [15], which over time may manifest as accelerated clinical deterioration or increased lesion burden, including new or enlarging T2 lesions and T1 gadolinium-enhancing lesions on magnetic resonance imaging (MRI).

Studies comparing the long-term clinical outcomes of MS patients with and without COVID-19 have produced mixed results. Some have found that symptoms like dyspnea, fatigue, and hyposmia do not differ significantly between MS patients with and without COVID-19 at 12 months [16], nor do disability trajectories, relapse rates, or disease-modifying therapy change at two-year follow-up [17]. A longitudinal analysis observed no significant differences in symptoms or disability progression in MS patients with and without COVID-19 [18]. In contrast, other reports suggest a possible acceleration of MS disease activity post-infection: Rahmani et al. noted an increased risk of expanded disability status scale (EDSS) progression after one year [19], and our own previous research demonstrated higher all-cause mortality and new-onset optic neuritis in COVID-19-exposed MS patients over three and a half years [20].

Fewer studies have investigated the long-term MRI progression of MS patients with and without COVID-19. One study found a higher risk of new gadolinium-enhancing T1 lesions at 12 months among COVID-19 patients compared to controls [19]. A longitudinal study detected no global acceleration of brain atrophy pre- to post-infection but observed potential parahippocampal volume loss [21]. Another study reported no differences in new or enlarging T2 lesions or enhancing T1 lesions between COVID-19 and control patients [17]. However, these investigations lacked either longitudinal (pre- and post-infection) MRI data or did not compare to a non-COVID control group, making it difficult to confidently attribute observed differences to COVID-19 as opposed to pre-existing factors or natural progression of disease.

To address these gaps, we examined the long-term radiological progression of relapsing-remitting MS among patients with and without confirmed COVID-19. Our diverse cohort came from real-world data obtained in clinical settings in the Montefiore Health System in the Bronx which was the hotspot of early pandemic and subsequent waves. By comparing pre- and post-index date MRIs that were 27 months apart on average, we determined whether SARS-CoV-2 infection influenced lesion burden and other imaging markers of MS progression.

## 2. Materials and Methods

### 2.1. Data Sources

This retrospective, longitudinal, single-center study was approved by the institutional review board with a waiver of informed consent. Data were retrieved from the electronic health records (EHR) system of the institution, covering the period from 1 January 2016 to 12 January 2024. This EHR system includes multiple hospitals and outpatient clinics serving the Bronx, NY, and surrounding regions. Data extraction was performed using the Observational Medical Outcomes Partnership (OMOP) common data model, as previously described [20,22,23,24,25,26,27,28,29,30,31,32,33,34,35,36]. Manual chart reviews were routinely conducted on selected patient subsets to validate key variables. The study consisted of real-world data obtained in clinical settings using standard-of-care and heterogeneous imaging and neurological evaluation protocols.

### 2.2. Study Cohort

Only patients with pre-existing relapsing-remitting MS at the index date were included (International Classification of Diseases, 10th Revision, G35). Among COVID-19 patients, the index date was defined as the date of the first positive SARS-CoV-2 polymerase chain reaction (PCR) test. Patients with at least one pre- and one post-COVID-19 brain MRI were selected. COVID− patients were those without a positive PCR test. Among 1170 COVID− patients, all visits that occurred after 1 March 2020 and in between two brain MRIs were identified. This cohort was then 1:2 index date matched to the 39 COVID+ cohort based on the index date. For each match, one visit for the COVID− patient was selected to serve as the index date, ensuring it occurred within a month (±30 days) of the corresponding COVID+ patient’s index date.

### 2.3. Variables and Outcomes

Demographic data included age at index date, sex, race, and ethnicity. Baseline variables included vaccination status against SARS-CoV-2 and pre-existing comorbidities, including hypertension (HTN), type-2 diabetes mellitus (T2DM), chronic obstructive pulmonary disease (COPD), cardiovascular disease (CVD; composite of myocardial infarction, congestive heart failure, and coronary artery disease), chronic kidney disease (CKD), liver disease, tobacco use, and obesity.

### 2.4. MRI Scoring

Clinical brain MRI examinations were assessed longitudinally at two different time points for each patient: a pre-index MRI, defined as the most recent scan obtained between two weeks and one year prior to the index date, and a post-index MRI, defined as a follow-up scan obtained at least two weeks after the index date. On average, the interval between pre- and post-index MRIs was 2.20 years for COVID+ patients and 2.28 years for COVID− patients. MRI examinations were performed as part of routine clinical care using multiple scanners and protocol versions over time. A physician with more than five years of experience in clinical radiology and imaging research and a board-certified neuroradiologist with more than 10 years of experience assessed all MRI studies and were blinded to cohort designation. Radiologists scored each study independently after images were downloaded from the Picture Archiving and Communications System, with discrepancies resolved through consensus discussion. Each study was scored for the absence or presence of enlargement of previous lesions, new lesions, T1 gadolinium-enhancing lesions, and significant brain parenchymal volume loss between time points, with the latter assessed qualitatively by two radiologists on the basis of visible interval reduction in parenchymal volume compared with prior imaging. The heterogeneity of imaging protocols precluded quantitative image analysis (such as image segmentation to extract lesion volumes).

### 2.5. Analysis and Statistics

Python (version 3.8.19) was used for data processing and statistical analysis. *p*-values less than 0.05 were considered statistically significant. For group comparison of categorical variables, the χ^2^ test was used and for group comparison of continuous variables, the independent *t*-test was used. Univariate and multivariate logistic regression were used to estimate the odds of experiencing radiological progression between COVID+ vs. COVID− patients.

## 3. Results

### 3.1. Cohort Selection

**Figure 1:**
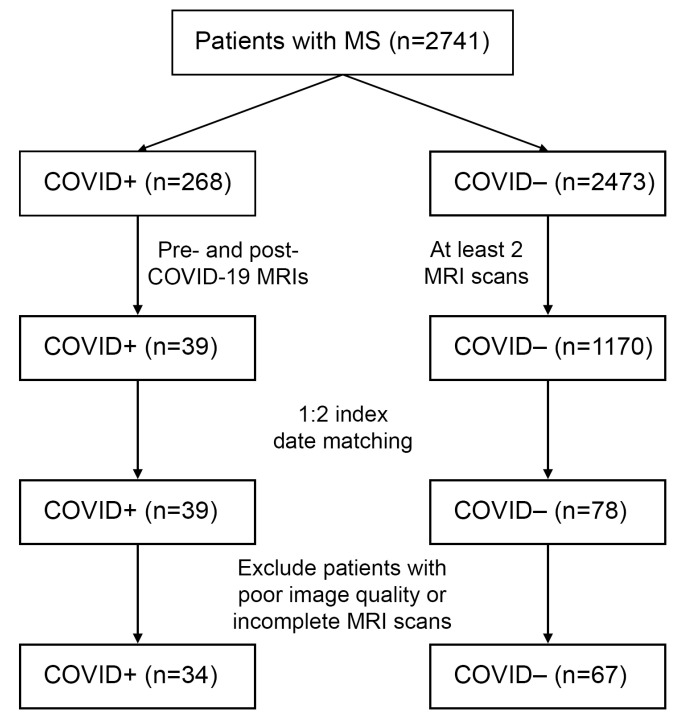
Patient selection flowchart. MS: multiple sclerosis.

Figure 1 shows the patient selection flowchart. Medical records of 2741 patients with pre-existing relapsing-remitting MS were screened for SARS-CoV-2 PCR tests from 1 February 2020 to 12 January 2024. 268 patients with pre-existing MS had a confirmed SARS-CoV-2 infection (COVID+), while 2473 had no recorded history of COVID-19 (COVID−). In the COVID+ group, 39 individuals had at least one brain MRI before and one after the index date. All visits occurring after 1 March 2020 and in between two brain MRIs were identified for the 1170 COVID− control patients, which were used as a matching pool for the 1:2 index date match. In 4 COVID+ patients, only one match was obtained, resulting in 4 fewer COVID− matches than expected (74 vs. 78). Finally, individuals with incomplete MRI series (e.g., missing sequences or insufficient quality) were excluded, leaving 34 COVID+ and 67 COVID− patients for subsequent analysis.

### 3.2. Baseline Characteristics

Baseline demographics and comorbidities were similar between the two groups (Table 1). The mean (±standard deviation) age (COVID+: 47.50 ± 14.66 years vs. COVID−: 47.52 ± 12.77 years), years between MRIs (2.20 ± 0.96 vs. 2.28 ± 1.11), and years from index date to latest MRI (1.78 ± 0.98 vs. 1.67 ± 1.02) were similar (*p* > 0.05). Years from MS diagnosis to index date were longer in COVID− patients (3.34 ± 1.87 vs. 4.35 ± 1.80, *p* = 0.0096). The proportion of female patients was similar in both groups (79.41% vs. 85.07%, *p* > 0.05). There were no group differences in comorbidities (HTN, T2DM, COPD, CVD, CKD, liver disease, tobacco use, and obesity) (*p* > 0.05). Prior history of cerebral infarction was present in 4 (11.76%) COVID+ patients and 5 (7.46%) COVID− patients (*p* > 0.05). No patients had a recorded history of intracranial hemorrhage or primary or secondary intracranial cancer. One patient in the COVID− group had a prior traumatic brain injury. A total of 61.76% of COVID-19 patients and 65.67% of COVID− patients were vaccinated for SARS-CoV-2 prior to the index date (*p* > 0.05), and 47.06% of COVID-19 patients were hospitalized during acute infection.

### 3.3. Radiological Outcomes

Overall, comparison of pre- and post-index date MRIs indicated no statistically significant differences in MS progression between the COVID+ and COVID− groups (Table 2).

**Figure 2:**
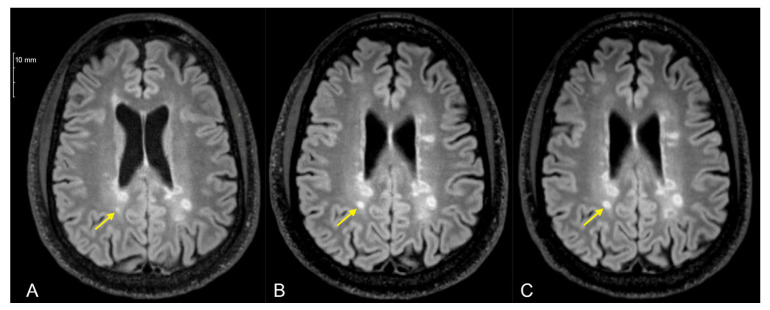
T2 fluid-attenuated inversion recovery magnetic resonance imaging of a patient at the (**A**) pre-index timepoint, (**B**) 21 months and (**C**) 46 months after the pre-index timepoint. A lesion in the white matter posterior to the body of the right lateral ventricle (arrow) appeared small in (**A**), measuring 1.55 × 1.50 mm and spanning over two axial slices. It gradually increased in size on two subsequent post-index scans, measuring 4.24 × 5.25 mm in (**B**) and 5.20 × 6.29 mm in (**C**) and spanning four axial slices. Note the slight variation in the axial plane angle among the three scans.

**Figure 3:**
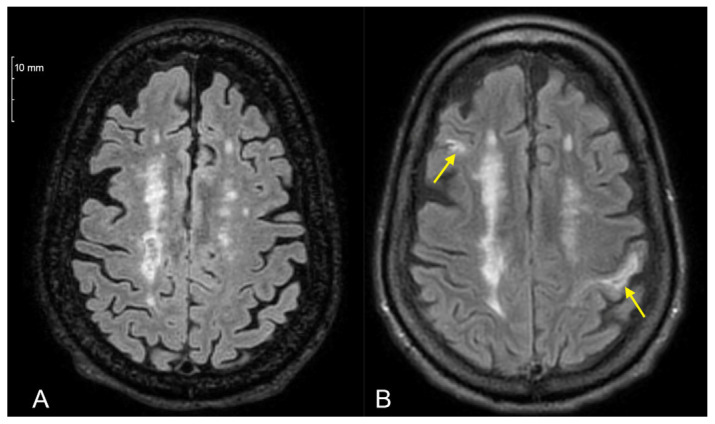
T2 fluid-attenuated inversion recovery magnetic resonance imaging of a patient (**A**) pre-index and (**B**) 18 months after pre-index date timepoint. The patient exhibited significant MS progression: apparent new lesions can be seen bilaterally in the left postcentral gyrus and right middle frontal gyrus (arrows).

Enlargement of previous lesions was observed in one COVID+ and 4 COVID− patients (2.94% vs. 5.97%, *p* = 0.86) (Figure 2). The occurrence of new lesions was infrequent and similar in both groups (3 [8.82%] vs. 6 [8.96%], *p* = 1.00) (Figure 3). Gadolinium-enhancing lesions were similarly rare (2 [5.88%] vs. 1 [1.49%], *p* = 0.54). Multivariate adjustment was not possible in the outcome of enhancing lesions due to low occurrence. Notable parenchymal brain volume loss was observed in 12 COVID+ and 32 COVID− patients (35.29% vs. 47.76%, *p* = 0.33).

## 4. Discussion

In this longitudinal assessment of relapsing-remitting MS patients with and without COVID-19 who underwent pre- and post-index date brain MRI, we did not detect differences in the occurrence of enlarging, new, or enhancing white matter lesions, or parenchymal brain volume loss. Our results indicate that, within our timeframe and population, there is no evidence that SARS-CoV-2 infection accelerates radiological progression of MS compared with uninfected controls; however, these results should be interpreted as exploratory given the qualitative nature of our MRI assessments.

The comparable rates of lesion enlargement, new lesion formation, gadolinium enhancement, and brain volume loss between COVID+ and COVID− groups align with recent evidence suggesting that SARS-CoV-2 infection does not trigger sustained MS disease activity [17,18]. The similar occurrence of new T2 lesions (8.82% vs. 8.96%, *p* = 1.00) and gadolinium-enhancing lesions (5.88% vs. 1.49%, *p* = 0.54) between groups mirrors findings from Montini et al., who reported comparable rates of new T2 lesions (9% vs. 11%, *p* = 1.00) and gadolinium-enhancing lesions (7% vs. 4%, *p* = 1.00) in 136 MS patients with COVID-19 compared to 186 matched controls over 18–24 months of follow-up [17]. The low frequency of enhancing lesions in both cohorts suggests minimal inflammatory activity, which is typical in treated MS populations.

The infrequent enlargement of previous lesions (2.94% vs. 5.97%, *p* = 0.86) further supports the absence of COVID-19-associated disease exacerbation. Slowly expanding lesions, particularly those with paramagnetic rims, have been associated with chronic active inflammation and disability progression [37]. The present findings suggest that COVID-19 does not accelerate this process of chronic lesion evolution.

We also found no group differences in parenchymal brain volume. This finding is supported by Devogelaere et al., who found that brain volume did not predict COVID-19 severity in 198 MS patients, and those with pre-existing atrophy showed cognitive decline related to their baseline pathology rather than COVID-19 itself [38]. In a cohort of 14 COVID-exposed patients with MS from Switzerland with longitudinal MRIs (average of 6.7 pre-infection MRIs and 1.4 post-infection MRIs), Rebsamen et al. reported no difference in pre- and post-infection global gray matter volume but did find lower parahippocampal gyri volume post-infection. Upon further investigation, this reduced parahippocampal volume was limited to unvaccinated patients who had not received SARS-CoV-2–specific treatment, whereas no such changes were observed in vaccinated patients and/or those who had received anti-SARS-CoV-2–specific treatment [21].

These results contrast with one study by Rahmani et al., which reported that COVID-19 was associated with significantly higher increases in MRI lesions (OR 6.37, *p* = 0.019) and new gadolinium-enhancing lesions over 12 months in 362 RRMS patients [19]. However, this finding has not been replicated in larger, more recent studies. The largest controlled interrupted time series analysis by Salter et al., involving 796 COVID+ MS patients followed for a median of 18 months, found no changes in symptom severity or disability trajectories after COVID-19 [18]. Similarly, Bsteh et al. reported that COVID-19 was not associated with increased risk of relapse (OR 1.1, *p* = 0.70) or disability worsening (OR 0.96, *p* = 0.60) in a nationwide multicenter matched-control study of 211 MS patients [39].

Discrepancies across the literature may reflect heterogeneity in patient populations, severity of COVID-19 disease, exposure to different viral strains across pandemic waves, and variation in vaccination status, follow-up duration, and MS treatment protocols. Publication bias, wherein null findings are less likely to be published, may also contribute to apparent inconsistencies in the literature. Additionally, our cohort was drawn from a diverse urban population in the Bronx with large proportions of Black and Hispanic patients, who are historically underrepresented in MS research and face significant health disparities and inequities in MS care [40].

Interpretation of MRI findings with respect to MS disease progression is complex, as white matter lesions may reflect overlapping processes, including demyelination, post-infectious inflammation, or microvascular injury. Imaging biomarkers such as the central vein sign, which improves specificity for MS lesions, may help distinguish true disease activity from mimickers [41]. Brain atrophy in MS is a gradual process, and detecting acceleration of volume loss may require longer observation periods and larger sample sizes. In addition, radiological findings may not fully represent clinical disease progression, as validated measures such as the Expanded Disability Status Scale (EDSS) provide a complementary assessment of functional outcomes. Beyond direct neuroinflammatory effects, COVID-19 may also influence disease trajectories through indirect mechanisms, including persistent systemic inflammation, reduced mobility, delayed rehabilitation, and disruptions in access to care or disease-modifying therapies. Increasing evidence suggests that prior infection can alter recovery patterns and clinical outcomes across conditions, supporting a broader role for systemic and functional factors in influencing long-term disease course [20,26,35,42].

The mechanisms by which viral infections might theoretically trigger MS disease activity have been studied, particularly in the context of Epstein–Barr virus (EBV), which has a strong epidemiological association with MS [43]. Proposed mechanisms include molecular mimicry, whereby structural similarities between viral and host proteins break immunological tolerance and trigger autoimmune responses, and direct viral-induced neuroinflammation through cytokine storm and immune dysregulation [13,43]. While case reports have documented individual instances of MS relapses temporally associated with COVID-19, systematic reviews suggest these events are relatively rare, given the widespread prevalence of SARS-CoV-2 infection [44,45,46,47]. The rate of CNS demyelinating events occurring in the setting of SARS-CoV-2 infection appears low, and clinical outcomes are generally favorable [46,48].

### Strengths and Limitations

To our knowledge, this study is among the first to longitudinally track MRI changes in MS patients before and after SARS-CoV-2 infection over an extended follow-up period of up to four years using real-world data, while comparing to a matched control group. A key methodological strength is that each patient’s follow-up MRI was directly compared to their pre-index scan, allowing more confident attribution of potential changes to SARS-CoV-2 infection rather than pre-existing differences or natural MS progression.

Several limitations of this study must be acknowledged. First, results need to be interpreted with caution, given the small sample sizes and the low frequency of radiological events, which limit statistical power. Second, our MRI analysis relied on a qualitative, binary assessment of new or enlarging lesions, enhancing lesions, and significant brain volume loss, without quantitative metrics. Subtle radiological progression may not have been detected because scans were obtained on different scanners using variable protocols. Third, we did not control for potential changes in MS therapies during this period, a major confounder given the impact of disease-modifying therapies on MRI activity. Additionally, we did not evaluate the association between COVID-19 treatments and outcomes due to the heterogeneity and inconsistent application of treatment regimens throughout the pandemic, particularly in its early stages [49]. Fourth, we were unable to meaningfully analyze differences in vaccinated or hospitalized subgroups due to the small sample size. Fifth, while our study extends up to four years post-infection, the effect of COVID-19 on radiological progression may be delayed and a longer follow-up time is needed. Finally, validated measures of MS disease severity, such as the EDSS, which are often not obtained in a clinical setting, were unavailable to us and thus remained unaccounted for in this analysis.

## 5. Conclusions

In this longitudinal study of patients with relapsing-remitting MS with and without COVID-19, we found no significant differences in qualitative radiological markers of disease progression. Future multi-center studies using standardized MRI protocols, quantitative lesion and brain volume measurements, and longer follow-up are warranted to confirm these exploratory findings.

## Figures and Tables

**Table 1:** Baseline characteristics of relapsing-remitting multiple sclerosis patients with and without COVID-19. None of the variables were statistically significant between COVID+ and COVID− group.

Characteristics	COVID+ (*n* = 34)	COVID− (*n* = 67)
Age at index date (years), mean ± SD	47.50 ± 14.66	47.52 ± 12.77
Years between earliest and latest MRI, mean ± SD	2.20 ± 0.96	2.28 ± 1.11
Years between index date and latest MRI, mean ± SD	1.78 ± 0.98	1.67 ± 1.02
Years between MS diagnosis and index date, mean ± SD	3.34 ± 1.87	4.35 ± 1.80 **
Female, *n* (%)	27 (79.41%)	57 (85.07%)
**Race and Ethnicity, *n* (%)**		
Non-Hispanic White	7 (20.59%)	5 (7.46%)
Black	15 (44.12%)	31 (46.27%)
Asian	0 (0.00%)	2 (2.99%)
Hispanic	13 (38.24%)	28 (41.79%)
Other race	12 (35.29%)	29 (43.28%)
Vaccinated for SARS-CoV-2 at index date, *n* (%)	21 (61.76%)	44 (65.67%)
Hospitalized due to COVID-19, *n* (%)	16 (47.06%)	−
**Comorbidities, *n* (%)**		
Hypertension	13 (38.24%)	32 (47.76%)
Type-2 diabetes	8 (23.53%)	14 (20.90%)
COPD	0 (0.00%)	1 (1.49%)
Cardiovascular disease	5 (14.71%)	11 (16.42%)
Chronic kidney disease	4 (11.76%)	9 (13.43%)
Liver disease	7 (20.59%)	8 (11.94%)
Tobacco use	17 (50.00%)	41 (61.19%)
Obesity	20 (58.82%)	45 (67.16%)
Cerebral infarction	4 (11.76%)	5 (7.46%)
Intracranial hemorrhage	0 (0.00%)	0 (0.00%)
Traumatic brain injury	0 (0.00%)	1 (1.49%)
Intracranial cancer	0 (0.00%)	0 (0.00%)

SD: standard deviation. MRI: magnetic resonance imaging. COPD: chronic obstructive pulmonary disease. ** *p* < 0.01 between COVID+ and COVID−.

**Table 2:** Outcomes comparisons in multiple sclerosis patients with and without COVID-19.

Outcome	COVID+ (*n* = 34)	COVID− (*n* = 67)	*p*-Value	OR [95% CI]	aOR [95% CI]
Enlargement of existing lesions	1 (2.94%)	4 (5.97%)	0.86	0.46 [0.05, 4.31]	0.38 [0.04, 4.00]
New lesions	3 (8.82%)	6 (8.96%)	1.00	0.95 [0.22, 4.07]	0.79 [0.17, 3.73]
Enhancing lesions	2 (5.88%)	1 (1.49%)	0.54	4.00 [0.35, 45.79]	−
Brain volume loss	12 (35.29%)	32 (47.76%)	0.33	0.60 [0.25, 1.41]	0.43 [0.14, 1.29]

OR: odds ratio. aOR: adjusted OR. aORs are shown with multivariate adjustment for age, sex, race, ethnicity, follow-up time since index date, duration of multiple sclerosis at index date, vaccination status, and pre-existing comorbidities. Multivariate adjustment was not possible in the outcome of enhancing lesions due to low occurrence and overfitting.

## Data Availability

The data presented in this study are available from the corresponding author upon reasonable request due to privacy and ethical restrictions.

## Abbreviation

The following abbreviation is used in this manuscript:

MS	Multiple sclerosis
